# Activity of crude extracts from Brazilian cerrado plants against clinically relevant *Candida* species

**DOI:** 10.1186/s12906-016-1164-3

**Published:** 2016-07-11

**Authors:** Amabel Fernandes Correia, Dâmaris Silveira, Yris Maria Fonseca-Bazzo, Pérola Oliveira Magalhães, Christopher William Fagg, Elton Clementino da Silva, Suelí Maria Gomes, Lenora Gandolfi, Riccardo Pratesi, Yanna Karla de Medeiros Nóbrega

**Affiliations:** Interdisciplinary Laboratory of Biosciences, School of Medicine, Darcy Ribeiro Campus, University of Brasília, CEP 70.900.910 Brasília, DF Brazil; Central Public Health Laboratory of the District Federal (LACEN-DF), Medical Biology Management, Center of Parasitology and Mycology, CEP 70.830.010 Brasília, DF Brazil; Laboratory of Natural Products, Faculty of Health Sciences, Darcy Ribeiro Campus, University of Brasília, CEP 70.900.910 Brasília, DF Brazil; School of Pharmacy, Ceilândia Campus, University of Brasília, CEP 72.220.900 Brasília, DF Brazil; Department of Botany, Institute of Biological Science, Darcy Ribeiro Campus, University of Brasília, CEP 70.900.910 Brasília, DF Brazil; Immunogenetic and Chronic-degenerative Diseases Laboratory, School of Medicine, Darcy Ribeiro Campus, University of Brasília, CEP 70.900.910 Brasília, DF Brazil; Department of Pharmaceutical Sciences, School of Health Sciences, Darcy Ribeiro Campus, University of Brasília, CEP 70.900.910 Brasília, DF Brazil

**Keywords:** *Candida* spp, Antifungal, Medicinal plants, Crude plant extract

## Abstract

**Background:**

Medicinal plants have traditionally been used in many parts of the world as alternative medicine. Many extracts and essential oils isolated from plants have disclosed biological activity, justifying the investigation of their potential antimicrobial activity. In this study, the in vitro antifungal activity of six Brazilian Cerrado medicinal plant species were evaluated against clinically relevant *Candida* species.

**Methods:**

The crude extract plants were evaluated against American Type Culture Collection (ATCC) standard strains of *Candida* spp*.* using disk diffusion method and determining the minimum inhibitory concentration (MIC). The chemical study results were confirmed by HPLC method.

**Results:**

All six plant species showed antifungal activity. Among the species studied, *Eugenia dysenterica* and *Pouteria ramiflora* showed significant inhibitory activity against *C. tropicalis* at lowest MIC value of 125 and 500 μg/disc, respectively. The *Eugenia dysenterica* also disclosed MIC value of 125 μg/disc against *C. famata*, 250 μg/disc against *C. krusei* and 500 μg/disc against *C. guilliermondii* and *C. parapsilosis. Pouteria torta, Bauhinia rufa*, *Erythroxylum daphnites* and *Erythroxylum subrotundum* showed activity against the yeast strains with MIC value of 1000 μg/disc. The chemical study of the most bioactive extracts of *Eugenia dysenterica* and *Pouteria ramiflora* revealed catechin derivatives and flavonoids as main components.

**Conclusions:**

All six evaluated plant species showed good antifungal potential against several *Candida* strains. However, *E .dysenterica* and *P. ramiflora* showed the higher inhibitory effect against the non-*albicans Candida* species. Our results may contribute to the continuing search of new natural occurring products with antifungal activity.

## Background

The genus *Candida,* that includes about 200 different species, is a common commensal microorganism of the human skin and mucosal surfaces. However, some *Candida* species are opportunistic pathogens, capable of causing superficial or systemic infections, mostly in humans, mainly when debilitated or immunocompromised [1]. The systemic infections due to *Candida* species are severe infections that can cause high morbidity and mortality [2]. The invasive *Candida* infection represents the most common infection in hospitals worldwide and has an attributable mortality of 35 % to 60 %, causing public health and economic impact, because of high healthcare cost and prolonged length of hospitalization [3, 4]. Among the pathogenic species, *Candida albicans* is the most common cause of invasive fungal infections in hospital settings, although a growing number of new cases from non-*Candida albicans* species have been detected. An even greater disparity occurred with *Candida glabrata*, which has been identified as the second most common cause of invasive fungal infections in North America and fourth in rank order in Latin America [5]. *Candida tropicalis* and *Candida parapsilosis* have shown increased prevalence worldwide, as rarer species such as *Candida guilliermondii*, *Candida pelliculosa*, *Candida kefyr*, *Candida rugosa*, and *Candida famata* have [6].

The limited number of antifungal agents presently available for the treatment of fungal infections support the need for more effective and less toxic treatment strategies in view of the progressive increase in the frequency of infections by non-albicans *Candida* species, the elevated resistance to antifungal drugs, and the relative toxicity of the antifungal drugs currently in use [7]. Besides, selection pressure due to the continuous exposure of the infectious agent to azoles seems to be the main cause behind the development of resistance to fluconazole that has been considered the drug of choice against *Candida* species. In spite of its fungistatic nature a prolonged use of this drug is not advisable considering the side effects, hence the increased demand for agents from natural resources as they could presumably act in a host friendly manner [8, 9]. Medicinal plants have traditionally been used in many parts of the world as alternative treatments to traditional medicine [10]. Many extracts and essential oils isolated from plants have been shown to exert biological activity justifying the investigation of their potential antimicrobial activity [11, 12]. Plants of the Cerrado, a tropical highland savanna covering almost 800,000 sq mi in the Midwestern region of Brazil have been broadly used in popular medicine [13]. This region is characterized by an enormous range of plants that have been the focus of several research reports [14, 15] including the screening of several extracts and essential oils with potential anticandidiasis activity [16, 17].

In line with these efforts to increasingly exploit the therapeutic potential of plants of this extremely biodiversity rich region in the present study, we tested the crude extracts of the following botanical species: *Eugenia dysenterica* DC. [*Hexachlamys macedoi* Legrand), *Pouteria ramiflora* (Mart.) Radlk*, Pouteria torta* (Mart.) Radlk, *Bauhinia rufa* (Bong.) Steud, *Erythroxylum subrotundum* A. St.-Hil and *Erythroxylum daphnites* Mart. for their antifungal activity against American Type Culture Collection (ATCC) of *Candida* species standard strains.

## Methods

### Plant material

Leaves of *Eugenia dysenterica* DC. (*Hexachlamys macedoi* Legrand), *Pouteria ramiflora* (Mart.) Radlk*, Pouteria torta* (Mart.) Radlk, *Bauhinia rufa* (Bong.) Steud, *Erythroxylum subrotundum* A. St.-Hil and *Erythroxylum daphnites* Mart. were collected from the Cerrado biome (the Brazilian savanna) in the city of Brasília (Federal District), located in the Midwestern region of Brazil, and its surroundings. Botanical identification was performed by Professors Suelí Maria Gomes and Christopher William Fagg. The voucher herbarium specimens were deposited at the Herbarium of the University of Brasília (UB) (Table 1). All necessary permits were obtained for the described field studies.

**Table 1:** List of plant species used in the study

Plant species	Family	Extract solvent	Voucher specimen
*Eugenia dysenterica* DC (*Hexachlamys macedoi* Legrand)	Myrtaceae	A	914 (UB)
*Pouteria ramiflora* (Mart.) Radlk	Sapotaceae	A, E, H	3671 (UB)
*Pouteria torta* (Mart.) Radlk	Sapotaceae	E,H	3674 (UB)
*Bauhinia rufa* (Bong.) Steud	Fabaceae	A, E, H	32144 (UB)
*Erythroxylum subrotundum* A. St.-Hil	Erythroxylaceae	A, E, H	2194 (UB)
*Erythroxylum daphnites* Mart	Erythroxylaceae	A, E, H	2193 (UB)

Crude extracts from Leaves: A aqueous, E ethanolic, H hexanic

### Extraction procedures

Leaves were dried at room temperature and subsequently powdered in a knife mill (Marconi Laboratory Equipment, Piracicaba SP, Brazil). The ethanol and hexane crude extracts were obtained by the following way: plant material (40 g) was macerated at room temperature for seven days (repeated for three times), first with hexane (2 L), followed by ethanol (2 L). After filtration, the solvents were removed under reduced pressure, using rotary evaporator Hei-Vap coupled to vacuum pump model D-91126 (Heidolph Instruments GmbH & Co KG, Walpersdorfer, Schwabach) and refrigerator model MX07R-20-HD2E (Heidolph Instruments GmbH & Co KG, Walpersdorfer, Schwabach) to keep the temperatures below 40 °C. The aqueous crude extract was obtained by infusion, using 400 g of plant material and distilled water (3 L). After filtration procedure, water was removed by lyophilization using Advantage Plus XL-70 coupled to vacuum pump model 2005SD and air compressor model 1NNE5 (SP Scientific, Warminster, PA, EUA).

### Fungal strains

American Type Culture Collection (ATCC) standard strains of *Candida guilliermondii* (ATCC 6260), *Candida tropicalis* (ATCC 28707), *Candida parapsilosis* (ATCC 22019), *Candida albicans* (ATCC 90028), *Candida glabrata* (ATCC 2001), *Candida famata* (ATCC 62894), and *Candida krusei* (ATCC 34135) were used to perform the antifungal activity. The strains were kindly provided by the Oswaldo Cruz Foundation (FIOCRUZ) and stored at −20 °C.

### Screening of antifungal effect

For the experiment, 1000 μg of each extract were dissolved in 1 mL of the respective solvent (hexane, ethanol and distilled water) yielding final stock solutions with a concentration of 1000 μgmL^−1^. Six millimeters filter paper discs were soaked with 10 μL of each extract stock solution hexanic, ethanolic and aqueous crude extracts and dried at room temperature. Agar Sabouraud 24 h cultures (35 ± 2 °C) of *Candida* species were used in the preparation of an inoculum diluted in a 0.85 % saline solution, obtaining a fungal suspension equivalent to a 0.5 standard on the McFarland scale (1 × 10^6^ to 5 × 10^6^ cells per mL). On Petri dishes the fungal inoculum was spread over the surface of Mueller-Hinton + glucose (2 %) + methylene blue (0.5 μgmL^−1^) agar medium in which discs with the extracts and controls were subsequently deposited. The reading of the zone of inhibition was taken after 24 to 48 h of incubation at 35 ± 2 °C [18, 19]. Fluconazole (25 μg/disc) was used as positive reference standard and, as negative control filter paper discs with ethanol, distilled water and hexane.

### Minimal Inhibitory Concentrations (MIC) test

Only extracts that disclosed inhibition zone against the different *Candida* species underwent the MIC evaluation in the disc-diffusion assay, this technique was adapted by Razmavar et al. [19]. The extract stock solution was twofold diluted by serial dilutions resulting in concentrations ranging from 500 to 15.6 μgmL^−1^. After the serial dilutions, 10 μL of each dilution was soaked into 6 mm filter paper discs that were subsequently dried at room temperature. The fungal inoculum was spread over the surface of Mueller-Hinton + glucose (2 %) + methylene blue (0.5 μgmL^−1^) agar and discs were deposited in the culture medium. The plates were incubated at 35 ± 2 °C for 24 to 48 h [19]. The MIC (minimum inhibitory concentration) was defined as the lowest concentration of the tested extracts that was able to inhibit any visible microbial growth [20]. Solvent free extract was used as negative control and Fluconazole (25 μg/disc) was used as positive reference standard.

### Apparatus and chromatographic conditions

The extracts were Analyzed using LaChrom Elite HPLC system (Hitachi, Japan) liquid chromatograph equipped with L2130 pump, L2200 auto-sampler; L2300 column oven was in set at 25 °C. The detector, L2455 DAD (Hitachi, Japan) was set at 280 nm. Separation was performed by Purospher Star reverse phase C18e column (5 μm, 150 mm × 4.6 mm i.d.) in combination with an appropriate guard column (4 × 4; 5 μm particle size) (Merck, Germany). The mobile phase was a linear solvent gradient system consisting of phosphoric acid (1 %) (A) and CH_3_CN (B), at a flow rate of 0.6 mL/min. Data acquisition was performed using EXChrom Elite software (version 3.3.2 SP1, Scientific Software. Inc.). The compounds present in the extract were characterized according to their UV–vis spectra and identified by their retention time in comparison with those of commercial standards (catechin, epicatechin, isoquercitrin myricetin, caffeic acid, chlorogenic acid, ferulic acid, kaempherol, rosmarinic acid, hesperentin, hesperidin, ellagic acid, gallic acid, hyperoside, quercetin, revesratrol, rutin, vitexin and isovitexin) [14].

## Results and discussions

The antifungal activity of crude extracts of *Eugenia dysenterica*, *Pouteria ramiflora*, *Pouteria torta, Bauhinia rufa*, *Erythroxylum subrotundum* and *Erythroxylum daphnites* against ATCC standards strains of *Candida* spp. are shown in Table 2. The ethanol extract of *Erythroxylum daphnites* and all the hexane extract failed to show any inhibitory activity. However, all aqueous extracts and the remaining five ethanol extracts showed a variable inhibitory activity against one or more species of *Candida*.

**Table 2:** Antifungal activity of ATCC strains exerted by plant crude extracts by the Embedded Disc Assay

Strain ATCC	Origin	Inhibition Diameter (mm)															
		Aqueous Extract					Ethanolic Extract					Hexanic Extract					
		*E. dysenterica*	*P. ramiflora*	*E. subrotundum*	*E. dapnites*	*B. rufa*	*P. ramiflora*	*P. torta*	*E. subrotundum*	*E. dapnites*	*B. rufa*	*P. ramiflora*	*P. torta*	*E. subrotundum*	*E. dapnites*	*B. rufa*	*Fluconazole 25 μg*
*C. glabrata* (2001)	Feces	-	20	30	24	25	23	15	20	-	25	-	-	-	-	-	40
*C. parapsilosis* (22019)	Feces	15	-	11	-	10	-	10	10	-	-	-	-	-	-	-	33
*C. guilliermondii* (6260)	Saliva	12	-	10	-	-	-	10	-	-	-	-	-	-	-	-	50
*C. tropicalis* (28707)	Urine	12	12	-	-	-	13	-	-	-	-	-	-	-	-	-	30
*C. krusei* (34135)	Clinical Specimen	13	-	-	-	-	10	-	-	-	-	-	-	-	-	-	18
*C. famata* (62894)	Catheter tip	12	-	-	-	-	-	-	-	-	-	-	-	-	-	-	35
*C. albicans* (90028)	Blood	-	-	-	-	-	-	-	-	-	-	-	-	-	-	-	39

(−) Absence of antifungal activity

Among all the extracts that were assessed the aqueous extract of *Eugenia dysenterica* was the most effective. Except for *C. albicans* and *C. glabatra* in which no inhibition could be detected, this extract showed significant inhibitory activity against *C. parapsilosis*, *C. guilliermondii*, *C. tropicalis*, *C. krusei* and *C. famata*, with a zone of inhibition extending from 12 to 15 mm. The ethanolic extract *Pouteria ramiflora* also showed significant antifungal potential against *C. glabrata, C. tropicalis* and *C. krusei,* disclosing a zone of inhibition varying between 10 to 23 mm. Similarly, the ethanol extract of *Pouteria torta* and the aqueous extract of *Erythroxylum subrotundum* were effective against three *Candida* species and showed comparable antifungal activity against *C. parapsilosis* (10 and 11 mm), *C. guilliermondii* (10 mm) and *C. glabrata* (15 and 30 mm). Two species, *C. tropicalis* (12 mm) and *C. glabrata* (20 mm) were inhibited by aqueous extract of *Pouteria ramiflora* while the ethanolic extract of *Erythroxylum subrotundum* and the aqueous extract of *Bauhinia rufa,* revealed antifungal activity against *C. parapsilosi*s (10 mm) and *C. glabrata* (20 to 25 mm). Finally, the aqueous extract of *Erythroxylum daphnites* and the ethanolic extract of *Bauhinia rufa* showed inhibitory effect only against *C. glabrata* (24 and 25 mm). In the present study, *C. glabrata* was the most sensitive specie to the inhibitory activity of the majority of the extracts, whereas *C. albicans* was the most resistant since none of the assessed extracts showed any effect on its growth. The other species of *Candida* showed variable degrees of resistance and sensitivity to different extracts that were tested.

Scarce previous reports exist on the antifungal activity of the medicinal plants hereby described. In support of our results Costa et al. [21] described absence of inhibitory activity of *E. dysenterica* essential oil against *C. albicans* although it showed effectiveness against the pathogenic yeast *Cryptococcus.* Boleti et al. [22] reported the antifungal potential of *P. torta* extract against different yeasts and filamentous fungi and considered that the action of the pouterin, a lectin-like protein found in its seeds, interacting with cell wall carbohydrates of the fungus, results in an inhibitory effect on the growth. As far as we know antifungal activity of *P. ramiflora, E. subrotundum*, *E. daphnites* and *B. rufa* has not be previously reported, although some studies have identified the presence of alkaloids and flavonoids in the genus *Erythroxylum* [23–25] which may partly explain its activity against *Candida*. Antifungal activity has also been reported for some species *Bauhinia* [26, 27].

In view of the scarcity of data regarding the antifungal activity against *Candida* yeasts of the plants tested in the present study and in order to identify and confirm the possible medicinal potential of the extracts that had previously disclosed positive results in an initial screening, i.e. extracts that had exhibited an inhibition zone ≥ 10 mm against *Candida* species, we proceeded to the determination of their final minimal inhibitory concentration (MIC).

The results of MIC values from the diverse plant extracts tested are shown in Table 3. The results confirmed that the aqueous extract of *E. dysenterica* was the most effective against the different species of *Candida*. This extract exhibited respectively a MIC 125 μg/disc against *C. tropicalis* and *C. famata,* a MIC of 250 μg/disc against *C. krusei* and a MIC of 500 μg/disc against *C. parapsilosis* and *C. guilliermondii*. On the other hand, the aqueous and ethanolic extract of *P. ramiflora* showed effectivity against *C. tropicalis* with a MIC of 500 μg/disc. A MIC of 1000 μg/disc was found on testing the remaining extracts.

**Table 3:** The Minimum Inhibitory Concentration (MIC) of ATCC strains of *Candida* spp. exerted by plant crude extracts by the Embedded Disc Assay

ATCC	Origin	CIM (μg/disc)															Diameter (mm)
		Aqueous Extract					Ethanolic Extract					Hexanic Extract					
		*E. dysenterica*	*P. ramiflora*	*E. subrotundum*	*E. dapnites*	*B. rufa*	*P. ramiflora*	*P. torta*	*E. subrotundum*	*E. dapnites*	*B. rufa*	*P. ramiflora*	*P. torta*	*E. subrotundum*	*E. dapnites*	*B. rufa*	*Fluconazole 25 μg*
*C. glabrata* (2001)	Feces	-	1000	1000	1000	1000	1000	1000	1000	-	1000	-	-	-	-	-	40
*C. parapsilosis* (22019)	Feces	500	-	1000	-	1000	-	1000	1000	-	-	-	-	-	-	-	33
*C. guilliermondii* (6260)	Saliva	500	-	1000	-	-	-	1000	-	-	-	-	-	-	-	-	50
*C. tropicalis* (28707)	Urine	125	500	-	-	-	500	-	-	-	-	-	-	-	-	-	30
*C. krusei* (34135)	Clinical Specimen	250	-	-	-	-	1000	-	-	-	-	-	-	-	-	-	18
*C. famata* (62894)	Catheter tip	125	-	-	-	-	-	-	-	-	-	-	-	-	-	-	35
*C. albicans* (90028)	Blood	-	-	-	-	-	-	-	-	-	-	-	-	-	-	-	39

(-) Absence of antifungal activity

**Fig. 1:**
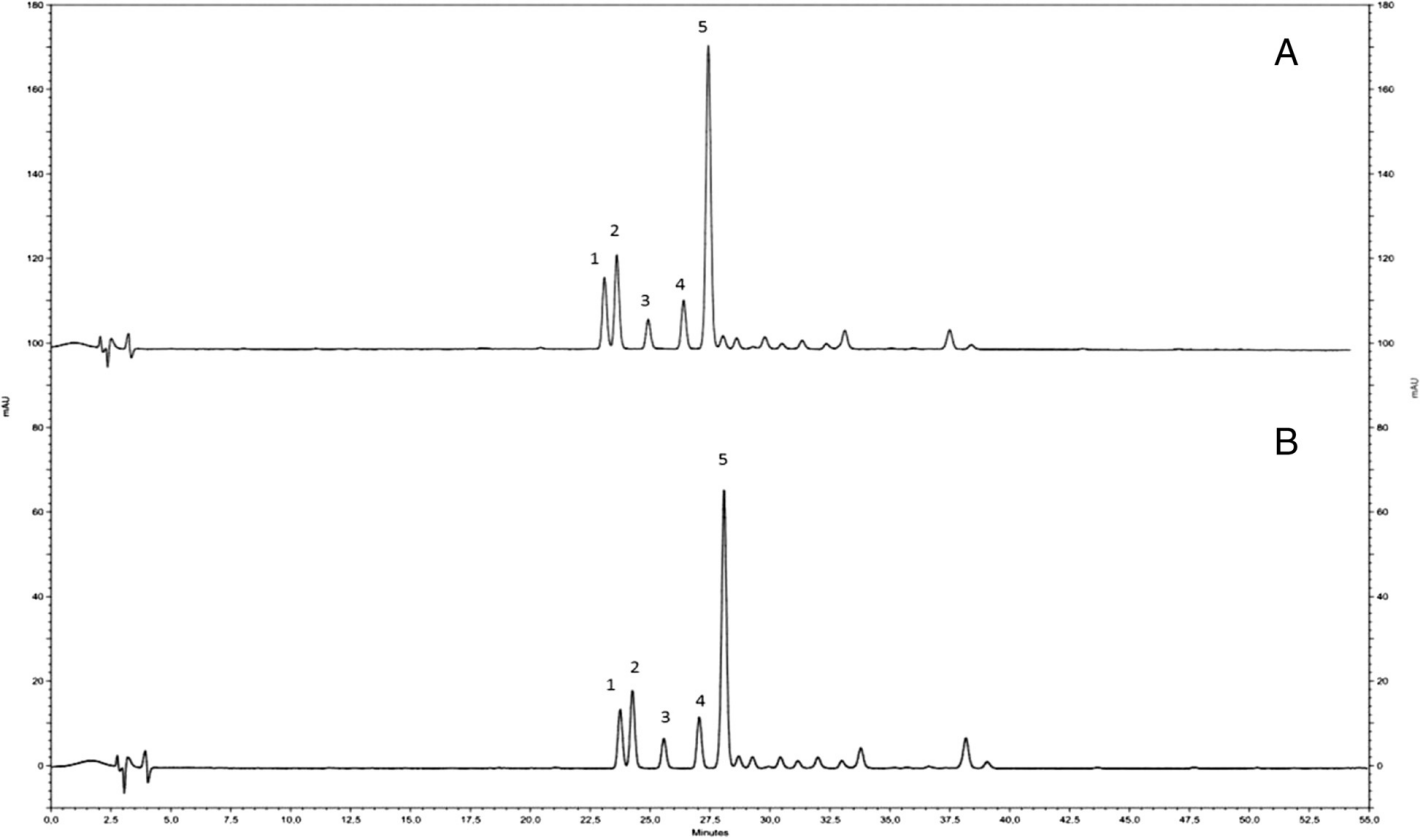
Chromatography profiles from aqueous and ethanolic leaf extracts from *Pouteria ramiflora*. **a** aqueous leaf extract from *Pouteria ramiflora* and **b** ethanolic leaf extract from *Pouteria ramiflora*

In a second step, considering that the lower the MIC of a drug, the greater its effectiveness, the phytochemical composition of *E. dysenterica* and *P. ramiflora* extracts, that had showed a MIC < 1000 μg/disc, was determined. The identification of the individual compounds responsible for the antifungal activity was performed by HPLC. The profiles of the aqueous and ethanolic extracts of *P. ramiflora* disclosed a large number of similar compounds with five main peaks (Fig. 1) [14]. The peaks 1–5 have characteristic UV/Vis spectra of flavonoids, with λ^max^ between 340 and 370 nm (Table 4). Identification of the peaks by comparison with commercial standards was not possible. Nevertheless, our *P. ramiflora* HPLC results showed similar UV/Vis peaks characteristic of the spectra of flavonoids as those found by Arapitsas [28]. Moreover, the peaks found in both extracts showed maximum absorption around 265 and 354 nm (Table 4) corroborating with maximum absorption of isorhamnetin derivatives described by Parejo et al. [29]. Flavonoids are the most studied groups of phenolic compounds, comprising molecules with established antifungal properties [30]. The study of compounds isolated from *Calotropis procera* Ait (*Asclepiadaceae*), ie the crude flavonoid fraction and flavonols (Quercetin-3-O-rutinoside, Kaempferol-3-O-rutinoside, Isorhamnetin-3-O-rutinoside, 5-Hydroxy-3,7-dimethoxyflavone-40-O-b-glucopyranoside), showed inhibitory effects of these compounds on *C. tropicalis* moreover against *C. albicans* [31].

**Table 4:** Retention time and maximum absorption of chromatographic peaks found in *P. ramiflora* extracts

Aqueous leaf extract from *P. ramiflora*		Alcoholic leaf extract from *P. ramiflora*	
Retention Time	Lambda max	Retention Time	Lambda max
23.84	265, 354	23.84	263, 357
24.35	263, 354	24.36	263, 354
25.65	268, 354	25.66	265, 358
27.13	265, 354	27.14	264, 358
28.23	262, 350	28.16	262, 350

**Fig. 2:**
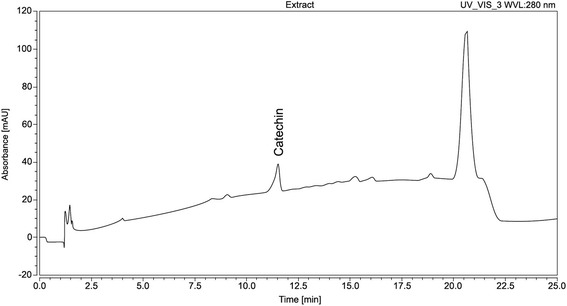
Chromatography profiles from aqueous leaf extracts from *Eugenia dysenterica*

On the other hand, the aqueous extract of *E. dysenterica* showed three main peaks, which have characteristic UV/Vis of catechin derivatives, with maximum absorbance near 280 nm and no absorption at 320 or 350 nm (Table 5, Fig. 2) [32]. Alves et al. [33] previously reported antifungal activity against species *Candida* of cathechin derivatives extracted from flowers of *Castanea sativa, Filipendula ulmaria, Rosa micrantha* and *Cytius multiflorus*, and from fresh leaves of *Cistus ladanifer*. The polyphenols (catechins and theaflavins) present in black tea also showed antifungal activity against species of *Candida* [34].

**Table 5:** Retention time and maximum absorption of chromatographic peaks found in *E. dysenterica* extracts

Aqueous leaf extract from *E. dysenterica*	
Retention Time	Lambda max (nm)
11.573	270
11.457	280
ND	300

ND (No Detected)

## Conclusions

All six evaluated plant species showed good antifungal potential against several *Candida* strains. *E. dysenterica* and *P. ramiflora* showed the higher inhibitory effect against the non-*albicans Candida* species. The chemical analysis of *E. dysenterica* and *P. ramiflora* extracts disclosed presence of polyphenols (flavonoids and catechins), an important chemical class with antifungal activity. Considering the increasing number of infections by non-*albicans* Candida species and focusing on their importance for public health, our results may contribute to the continuing search of new natural occurring products with antifungal activity.
